# The Double-Edged Sword of Erythrocytes in Health and Disease via Their Adhesiveness

**DOI:** 10.3390/ijms241210382

**Published:** 2023-06-20

**Authors:** Robert J. Asaro, Elisabetta Profumo, Brigitta Buttari, Pedro Cabrales

**Affiliations:** 1 Department of Structural Engineering, University of California, La Jolla, CA 92093-0085, USA; 2 Department of Cardiovascular and Endocrine-Metabolic Diseases, and Aging, Istituto Superiore di Sanità, 00161 Rome, Italy; elisabetta.profumo@iss.it (E.P.); brigitta.buttari@iss.it (B.B.); 3 Department of Bioengineering, University of California, La Jolla, CA 92093-0085, USA; pcabrales@ucsd.edu

**Keywords:** RBC adhesiveness, RBC hemolysis, RBC ghosting

## Abstract

Their widespread presence throughout the vasculature, coupled with their reactivity, and thereby to their potential to release reactive oxidative species, or to utilize their anti-oxidative capacities, has promoted much discussion of the role(s) of red blood cells (RBCs) in the progression of health or, alternatively, a wide range of disease states. Moreover, these role(s) have been linked to the development of adhesiveness and, in fact, thereby to the essential pathway to their eventual clearance, e.g., by macrophages in the spleen. These disparate roles coupled with the mechanisms involved are reviewed and given. Following an analysis, novel perspectives are provided; these perspectives can lead to novel assays for identifying the potential for RBC adhesiveness as suggested herein. We describe this paradigm, that involves RBC adhesiveness, hemolysis, and ghost formation, with examples including, inter alia, the progression of atherosclerosis and the suppression of tumor growth along with other disease states.

## 1. Introduction

A recent series of studies has explored a paradigm by which RBC adherence, under modest shear flows, leads to RBC vesiculation, hemolysis, and ghost cell formation [1,2,3]. This paradigm was directly linked to the basic physiology of RBC clearance, as for example in removal of senescent RBCs in the human spleen and most probably in the spleens of most invertebrates. Effectively, this rendering of the paradigm states that when senescent RBCs in the spleen become adherent to substrates such as the splenic endothelium, due to the exposure of adherence molecules, under even modest shear flows as exists there as in the vasculature, they tend to undergo vesiculation, hemolysis, and ghost cell formation. This is, in essence, what Klei et al. [4] proposed as a pathway for red cell splenic clearance and was mechanistically forecasted and thereby coincidentally explained by Asaro et al. [1] for splenic sequestering, vesiculation and possible lysis. This paradigm was developed by demonstrating how adhesion in shear flow drives the pathway for adhered cells to vesiculation, hemolysis, and ghost cell formation [2]. Moreover, the process was linked to the effects of Ca^++^ uptake and this suggested that the same basic pathway may be undertaken by RBCs without shear flow but with Ca^++^ uptake that persists for sufficiently long time periods. This was demonstrated by reviewing the results of osmotic fragility tests performed over time periods greater than 2–3 h and with biochemically induced Ca^++^ uptake [5]. Red blood cell vesiculation, hemolysis, and ghost formation, occurring outside the spleen, however, causes the release of heme, Fe^++^, and additionally of reactive oxygen species (ROS). This hence contributes to inflammation and oxidative stress and may precipitate reactions such as those leading to ferroptosis [6]. RBC vesiculation, hemolysis, and ghost formation would also lead to injection of membrane fractions that lead to calcification of atherosclerosis plaques and eventual plaque instability [7]. Both types of effects of RBCs are discussed below. An overall perspective on how RBCs participate in such disease progression is provided next. 

### Perspectives on the Red Blood Cell in Disease

Although generally RBCs are not considered adhesive cells [8], there are a wide range of studies that have demonstrated and characterized RBC adhesiveness to various substrates [9,10,11,12,13,14,15,16] with specific adhesion receptor molecules and their substrate ligands, e.g., to endothelial cells [13,16,17]. That is, when RBCs are subject to various stimuli, e.g., ROS especially under hypoxia as in disease states, they express a large number of adhesion receptors [15] that, in turn, can bind to substrate ligands also stimulated by the local oxidative disease state environment. Examples include α4β1 integrin [18,19], also known as the very late activation antigen 4 (VLA-4), 4–6 thrombospondin (TSP) receptor CD36 as well as Lu/BCAM activated in aged RBCs in the spleen [16]. Exposure of these molecules on the erythrocyte surface endows the cell with the propensity of adhering to the vascular endothelium [20,21]. Under such conditions, the RBC has been implicated in the progression of a range of disease states [1,3,7,22,23,24,25,26,27,28,29,30,31]. It has also been reported that human RBCs are stimulated to expose adhesion molecules by Ca^++^ uptake [32], a theme that was explored in detail [2].

RBCs are highly differentiated cells devoid of nuclei and other organelles; the evolution of this composition was designed to allow for the RBC’s large concentration of hemoglobin (Hb) that transports O_2_ to other cells and peripheral tissue [8]. However, extracellular Hb, released e.g., via RBC vesiculation or hemolysis, is a source of oxidative stress via the production of ROS, an effect briefly yet eloquently discussed by Rifkind et al. [33] who describe how the autoxidation of circulating Hb determines the formation of superoxide and H_2_O_2_ with Fe(II)-Hb, causing the production of highly reactive hydroxyl radicals. The oxidative effects of Hb and ROS have also been discussed by Nagy et al. [34] in connection with the progression of atherosclerosis plaques; in particular, they discuss how the prooxidant plaque environment promotes RBC lysis, hemoglobin oxidation, the release of heme and Fe^++^, leading to plaque instability.

With this perspective, we discuss manifestations of the paradigm by Asaro et al. [1,2,3] in two contrasting disease scenarios, viz. the progression and possible destabilization of atherosclerosis plaques [22,34] versus the decrease in cancer tumors [35]. In both cases we discuss how the promotion of red cell adhesion with even modest shear flows, e.g., with shear stresses in the range τ = 0.5–4.0 dynes cm^−2^ (~0.05–0.4 Pa), promotes vesiculation–hemolysis–ghost formation. Indeed, such RBCs tend to be of a senescent phenotype that is associated with adhesiveness.

## 2. Red Blood Cell Adhesion and the Pathway to Hemolysis

**Figure 1:**
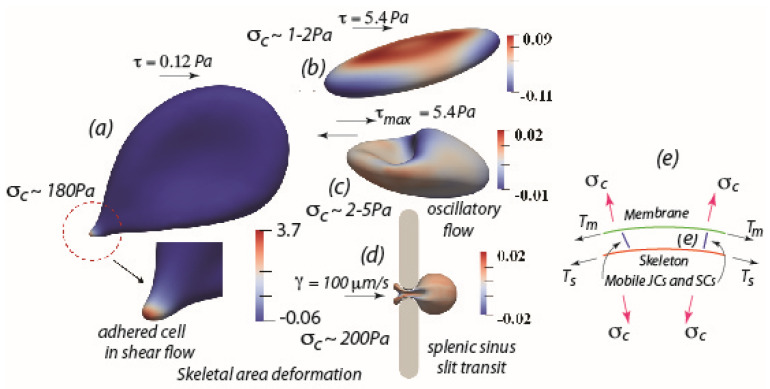
(**a**) An adhered RBC subject to a modest shear flow stress of τ~0.12 Pa (1.2 dynes/cm^2^) showing a large skeletal area expansion and a large contact stress δ_c_. (**b**,**c**) RBCs under laminar shear flow with sustained shear stress τ~5.4 Pa (54 dynes/cm^2^), (**b**), and oscillatory shear flow, (**c**), with an amplitude τ_max_~5.4 Pa (54 dynes/cm^2^), both showing very modest skeleton area deformations or contact stresses. (**d**) RBC flowing through the venous sinus slit of the human spleen, showing very modest skeleton area deformation and only a very short time period (<0.1–0.2 s) of contact stress, δ_c_. Note the transient nature of the large deformations preclude skeletal area changes. (**e**) Schematic of the RBC membrane with attached skeleton; note the differences in membrane tension, T_m_ and skeleton tension, T_s_, induce a contact stress, δ_c_ that, if positive, tends to separate the skeleton–membrane connection.

Red blood cell lysis occurs in an osmotic fragility test (OFT) essentially as the cell becomes spherical with decreasing relative tonicity (RT). This is readily understood by noting that once spherical, and since the cell membrane is very nearly area preserving, further decreases in RT will induce extreme increases in osmotic pressure and thereby increases in membrane tension; large membrane tension induces pores that lead to hemolysis. These effects are intensified by applied shear as, of course, exists in shear flow. Yet to appreciate the effects of applied shear forces it is necessary to recall that the RBC membrane is supported by a spectrin tetramer skeleton that is anchored to the bilipid membrane at two principal pinning complexes, both through mobile transmembrane proteins; these are: (1) the GPC/GPD/Rh/Duffy–actin–spectrin junctional complex (JC) via protein 4.1R; and (2) the so-called suspension complex (SC) involving band 3 with ankyrin. These pinning connections are indicated in Figure 1e where the RBC membrane is shown under tension T_m_ and its supporting skeleton under tension T_s_. Both transmembrane complexes are mobile and hence are subject to viscous drag motion. This mobility can result in large reductions in areal density of skeletal protein pinning points and a consequential loss in skeletal–membrane cohesion. That is, while the membrane area is fixed, the skeleton may expand area-wise so as to alter the areal density of pinning points via a reduction in the areal density of pinning points and a consequent loss in skeletal cohesion. Losses in skeleton–membrane cohesion favor tether, evagination, and vesiculation [36,37,38]. We refer to this process as skeleton remodeling. Hence, how applied shear forces may affect the tendency of a red cell to form tethers, vesicles, or eventually lyse, depends vitally on the scenario of the flow field vs. the boundary constraints imposed on the cell; this depends on, inter alia, how the cell is constrained, for example by adhesion to a substrate as opposed to being in free flow. It was precisely these effects that were studied by Asaro et al. [1] who first reported on the fundamental differences between the RBC skeletal remodeling and development of membrane tension in “adhered cells in shear flow” as compared to cells subjected to free flow, such as laminar shear flow, oscillatory shear flow (i.e., free shear flow of alternating sign), or short time transient flow such as flow through the fenestrations of the splenic venous sinus. They pointed out that such important events within the membrane–skeleton deformation are time dependent and characterized by distinct time scales; hence events such vesiculation, lysis, and subsequent ghost formation require time to occur. They further discussed the biochemical pathway to ghost cell formation, also characterized by various time scales that lead to skeletal–membrane decohesion that intensify membrane tension and promote lysis leading to ghost formation [2]. Simulations, such as shown in Figure 1a–d, are discussed in detail elsewhere [1,3] and here we provide a brief overview for perspective.

Figure 1a shows an adhered RBC subject to a quite modest shear flow stress of τ~0.12 Pa (1.2 dynes/cm^2^); the salient features under the conditions of cell adherence are the very large skeletal areal expansions measured by an areal strain A = λ_1_λ_2_ − 1, where λ_1_, λ_2_ are the two in-plane stretches of the skeleton, that is seen to reach magnitudes in the range 3.7; this range represents an essentially 370% expansion of the skeleton that results in over a factor of 10 reduction in the density of pinning points. This is coupled to a large so-called contact stress, δ_c_~180 Pa; the contact stress, depicted in Figure 1e, results from a difference in membrane/skeleton tensions and when positive, induces separation of the membrane–skeleton. It is these effects, particularly characteristic of adhered cells, that lead to skeletal–membrane separation, large membrane tension, and consequent effects such as Ca^++^ uptake and membrane shedding and lysis. On the other hand—and what results in a near absence of such effects—Figure 1b,c show RBCs in either free laminar shear flow (Figure 1b) or free oscillatory shear flow (Figure 1c) but under much higher shear stress, in this case τ~5.4 Pa, or for oscillatory flow τ_max_~5.4 Pa. What is found in such cases is a near vanishing of skeletal area expansions and very low contact stresses. In Figure 1d the case of RBC flow through a 1µm caliber slit is modeled after human splenic sinus slits [1]. Despite the obvious large deformations, especially near the so-called “in-folded” regions at the cell’s tail, the skeletal areal deformations remain vanishing small. The reason for this is that, since the time scale of such splenic passage is on the order of 0.1–0.2 s as measured via the videomicroscopy of MacDonald et al. [39], Asaro et al. [1] thereby found that there was insufficient time for viscous drag of the pinning points to cause measurable skeletal deformation. This again emphasizes the importance of cell adherence along with the importance of time of adherence under shear flow.

Taken together, these results explain why when cells are adhered under even quite modest shear flow for sufficient time, they tend to first demonstrate vesiculation, membrane shedding, lysis and ghost formation. This we hypothesize is a basis for explaining the effects that adherent RBCs play in disease progression. Such scenarios are discussed next.

## 3. Red Blood Cell Adhesive Interactions in Disease

RBC adhesive interactions have been implicated in the progression of a wide range of disease states where their roles have been specifically linked to their adhesiveness. Such diseases include, inter alia, atherosclerosis and the resulting thrombosis that may be caused, polycythemia vera, central retinal occlusion, sickle cell anemia, diabetes mellitus, Gaucher disease, and malaria [3,8,12,13,14,17,24,25,26,31]. In what follows, two examples of red blood cell effects, viz. on atherosclerosis and-perhaps as may seem counterintuitive-on tumor suppression are discussed, followed by briefer discussions of the role of adhered RBCs in other diseases, and finally the curious role RBCs play in the interactions with macrophages [40].

### 3.1. The Role of the Red Blood Cell in the Progression of Atherosclerosis

**Figure 2:**
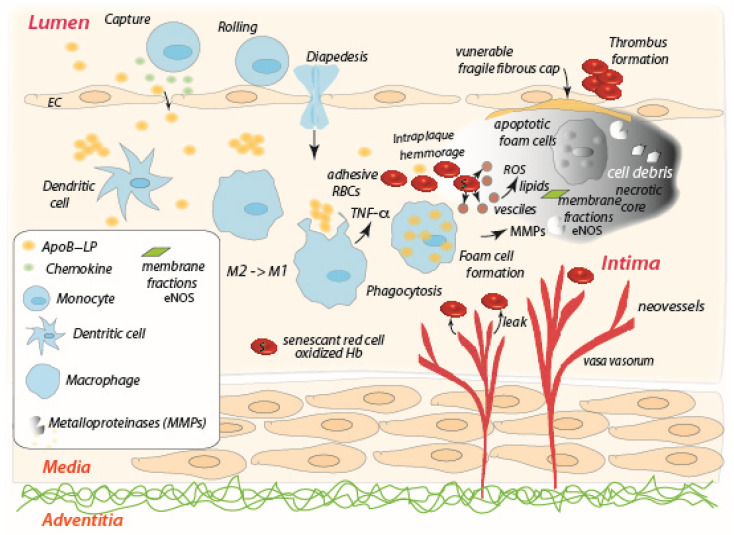
The interior of an atherosclerosis plaque characterized by hypoxia and unresolved, and intensifying, inflammation; the progression of the plaque development is from the upper left diagonally to the lower right and upward toward the necrotic core at the upper right. Extravasated RBCs become senescent-like, adherent, and may undergo vesiculation, hemolysis, thus producing ROS, shed membrane patches (i.e., membrane fractions), leading to calcification [7] and eventual plaque instability.

There is general agreement that the key initial step in atherosclerosis is the subendothelial retention of cholesterol rich apolipoprotein B-containing lipoproteins (apoB-LPs) in susceptible regions of non-laminar flow [41,42], see Figure 2 upper left. Retention of apoB-LPs by proteoglycans results in apoB-LP aggregates [43] and increases the susceptibility of apoB-LP oxidation [44]. A key inflammatory response to retained apoB-LPs that are prone to oxidative modification is activation of the overlying endothelial cells that leads to chemotactic recruitment of monocytes [45], see Figure 2 upper left. Accumulation of macrophages within the early plaque contributes to the secretion of apoB-LP-binding proteoglycans that further encourages LP retention; the excessive lipid accumulation in macrophages and vascular smooth muscle cells (VSMC) leads to the formation of foam cells that, in turn, contribute to the physical bulk of developing plaques by triggering multiple pathways of programmed cell death, including apoptosis, autophagy, necroptosis, and pyroptosis. All these events result in a failure of such lesions to resolve as depicted in Figure 2 [46,47,48].

The fate of atherosclerosis plaques is highly dependent on the balance between the recruitment and activation of monocyte-derived macrophages, and their clearance from the vessel wall [49,50,51,52,53,54,55]. Macrophages are extremely plastic cells, which quickly react in response to injury, infection, and other types of noxious conditions such as hypoxia and metabolic stress by switching phenotype to fulfil a pivotal role in host defense, wound healing, and immune regulation [56]. Macrophages with different phenotypes are, hence, distributed in varying locations of the atherosclerosis plaque [57].

After stimulation by microenvironmental factors, these cells demonstrate different polarization states [58]. In human atherosclerotic plaques, macrophages with a proinflammatory phenotype, termed M1, are aggregated on the shoulders of vulnerable plaques and promote inflammation by secreting proinflammatory cytokines, whereas macrophages with an anti-inflammatory phenotype, termed M2, are present in a stable cell-rich region far from the lipid core. M2 macrophages resolve plaque inflammation by secreting anti-inflammatory cytokines and by stimulating angiogenesis and phagocytosis. Macrophages termed Mhem, a more recently identified phenotype [59], are found accumulated in regions of previous hemorrhage and show an anti-atherogenic effect via the reduction of oxidative injuries in human plaque. These cells express high levels of scavenger receptor CD163, thus being able to clear Hb more quickly and to reduce hydrogen peroxide and ROS release [59]. In a mouse model of atherosclerosis, the macrophages in early-stage (“fatty-streak”) plaques are of the M2 type, but as the plaque progresses in size and complexity, they become M1-like [60].

Among microenvironmental factors, hypoxia plays a proatherogenic role in macrophage lipid and glucose metabolism, inflammation and polarization, and especially in the necrotic core where foam cells may undergo apoptosis [42] and where plaque destabilization occurs [61]. In addition, oxygen deprivation within the plaque induces neovascularization via the vasa vasorum [61,62], as indicated in Figure 2. Causes of hypoxia include, inter alia, the high metabolic needs involved with foam cell formation, as mentioned, reduced blood flow due to intima thickening, and generally reduced blood flow through the existing vasa vasorum. Hence the adaptive response is the growth of the vasa vasorum toward the lumen to supply oxygen to the inner layers [63]; however, intraplaque neovessels tend to be fragile, i.e., leaky, and prone to hemorrhage [62], mainly due to metalloprotease (MMP) activity as well as plasminogen activators [64]. Intraplaque hemorrhage is believed to be a primary trigger of the breach in the fibrous cap of the necrotic core that releases thrombosis [65,66].

Hence, we envision red blood cells entering into the unresolved hypoxic, inflamed, plaque environment; assuming RBCs are not yet senescent, the plaque environment clearly induces them to become so. The oxidative stress of the plaque will, for example, promote Ca^++^ uptake, transient cell shrinkage and re-swelling as described above and as described specifically by Lang et al. [9] for red blood cell “death”. Senescent RBCs are characterized by the exposure of phospholipid phosphatidylserine (PS) and adhesive receptors [67,68], and so we thereby hypothesize that the RBCs entering the plaque via hemorrhage become adhesive, and this is due to the inflammatory activity exerted by M1-like macrophages and hypoxia that promotes exposure of PS receptor (PSR) on the endothelial cells as described by Setty and Betal [15]. Under such circumstances RBCs will tend to undergo vesiculation and hemolysis, release Hb and create additional ROS as for aged RBCs [69], thus further exasperating the inflammatory conditions within the plaque. This is, in fact, quite consistent with the general scenario suggested by Buttari et al. [22] but, as yet, with an unknown mechanistic pathway; our paradigm is aimed at providing that necessary understanding. Buttari et al. [22] discuss a variety of RBC interactions involving cross-talk with T helper (Th1) cells, dendritic cells, and thereby contributing to the inflammatory factors that promote macrophage polarization toward the M1 inflammatory phenotype, that further supports our hypothesis. In particular, Buttari et al. observed that RBCs obtained from patients affected by carotid atherosclerosis failed to control lipopolysaccharide-induced dendritic cell (DC) maturation, thus determining the DC maturation toward a DC profile that sustains proinflammatory Th1 cell response [70]. Furthermore, Profumo et al. [71] observed that RBCs from patients with atherosclerosis are not able to inhibit activation-induced T lymphocyte apoptosis as opposed to RBCs from healthy subjects. As an impairment of apoptotic cells phagocytosis has been hypothesized as a mechanism contributing to necrotic core formation and to plaque vulnerability, it can also be speculated that the loss of the ability to rescue T lymphocytes from activation-induced apoptosis by RBCs exposed to oxidative stress contributes to promoting the pro-atherogenic processes responsible for plaque destabilization. These results indicate that the crosstalk between RBCs and immune cells contributes to the promotion of a proinflammatory microenvironment characterized by M1-like macrophage accumulation within the atherosclerosis plaque, and represents an additional pathway through which oxidative stress contributes to the progression of atherosclerosis. The M1-like macrophages increase is also supported by Williams et al. [72] who noted that “with plaque progression, an increase in GM-CSF expression in vivo contributes to the observed increase in M1-like cells”. We refer to their discussion for further details [22,72].

Finally, we mention that extravasated erythrocytes arising from intraplaque hemorrhage take on a senescent-like phenotype due to the hypoxic plaque environment and its ROS composition. They are likely adherent and hence prone to lysis as described in the above paradigm and its pathway. As shown by Tziakas et al. [7], the membranes of such lysed RBCs-but not intact human erythrocytes or lipids derived therefrom-promote mineralization of human arterial smooth muscle cells. This leads to plaque progression and eventual plaque instability via increased tissue stress [73]. The mechanisms involve lysed erythrocyte membranes inducing osteoblastic differentiation and calcium deposition mediated by eNOS activity via the NO receptor [7].

Collectively, all these observations indicate that exposure of RBCs to oxidative stress alters structural and functional features of these cells that consequently may acquire a proinflammatory behavior, thus contributing to the worsening of the atherosclerosis disease state.

### 3.2. The Role of the Red Blood Cell in Tumor Necrosis

**Figure 3:**
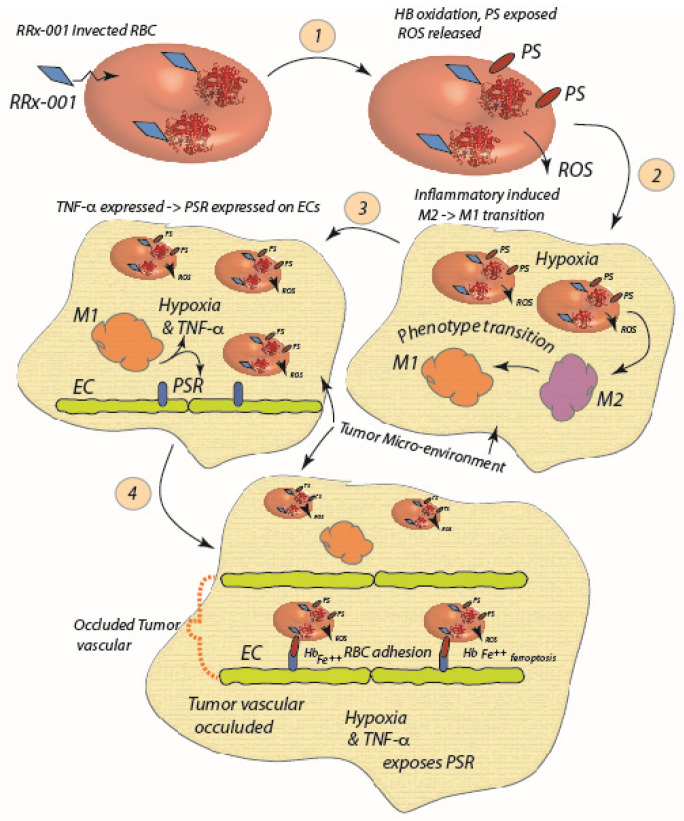
RRx-001 enters the red cell and binds to the Cys93 residue of, and oxidizes, Hb (1). The ROS released by now senescent RBCs induce a M2 to M1 macrophage polarization (2), and subsequent increase in TNF-α and hypoxia further exposes PSR and enhances RBC adhesion (3). RBCs undergo further hemolysis and contribute to the hypoxic, inflamed state of the tumor environment; heme and Fe^++^ released serve as endogenous sources of F^++^ and heme to promote ferroptosis (4) [6].

Recent studies provide evidence that adhered RBCs within tumor environments can lead to tumor mass reduction. The pathway for this was proposed as depicted in the scheme of Figure 3 and involves the minimally toxic pleiotropic anticancer agent RRx-001 [35,74]. RRx-001 treatment of human RBCs induces a senescent-like state in RBCs that promotes adhesiveness via the exposure of PS; the mechanism for this involves RRx-001 binding to the Cys93 residue of hemoglobin (Hb), and inducing oxidation of Hb that then stimulates exposure of PS to the outer bilipid leaflet, as occurs for instance in sickle cell disease [17,75] and in the vesiculation and clearance of senescent red blood cells. In the inflammatory, *viz.* hypoxic and acidic, state of the tumor environment, this leads to the release of ROS and the exposure of PSR, the receptor for PS [15]. Once adhered in even modest shear flow, RBCs may release oxidized Hb and ROS via the pathway described in Figure 3.

The environment so created favors the antitumor M1 macrophage phenotype and TNF-α release, that leads to further exposure of PSR, RBC adhesion, and hemolysis [35]. Moreover, it was shown that RRx-001 induces a surface decrease of CD47 on tumor cells and SIRP-α blocking on macrophages and a reduction of the phagocytosis inhibitory effect of the CD47/SIRP-α pathway that leads to a decrease in tumor mass, involving a shift from M2 macrophage to the more anti-tumor M1 phenotype [76,77,78,79,80]. It may be that RRx-001-treated RBCs stimulate a repolarization of tumor-associated-macrophages (TAMs) to a more M1-like phenotype via contributing to oxidative stress in the tumor environment and perhaps in a yet unrecognized molecular manner [79,80]. While ROS promotion of M1 polarization has been recognized and described, it is not clear if such favored polarization is exclusive to M1- vs. M2-like phenotypes [81]. For example, Zhang et al. [82] reported that ROS, and in particular O^2−^, play a “vital” role in the differentiation of the M2 phenotype, but not the M1 phenotype, where O^2−^ played little role; in fact by applying ROS inhibitors they demonstrated M2 differentiation was inhibited. This is consistent with the findings of Oršolić et al. [83] who also found that ROS production was “necessary for the differentiation of M2 macrophages”; yet they also noted that “ROS production was critical for the activation and functions of M1 macrophages”. Oršolić et al. [83] used the antioxidant caffeic acid to demonstrate blockage of differentiation of TAMs, i.e., in their view M2 phenotypes. A recent review by Virág et al. [84] has noted results, viz. those of Regdon et al. [85] that M1 polarization increases macrophage resistance to oxidative stress as a self-protective measure; the same review, however, points to the results of Dai et al. [86] that find that the M2 phenotype has increased resistance to lipid-mediated stress. On the other hand, it has been shown that free heme, a product of hemolysis of senescent RBCs, induces necrosis in macrophages and hence may result in a decrease in pro-tumor M2-like macrophages vs. the anti-tumor M1 phenotype, or a general decrease in all TAM phenotypes. Yet again, it has been reported that ROS may cause apoptosis in cancer cells [87,88] and hence the free heme released by RRx-001 affected RBCs along with the increased ROS generated may also contribute to cancer cell death.

On the other hand, the release of free heme and Fe^++^ during hemolysis, along with the earlier release of vesicles exposing PS, of senescent-like RBCs adhered in clusters to tumor cells may precipitate ferroptosis as described as affecting survival of cancer cells [6,89,90,91,92]. Following this hypothesis, we note two possibilities for more specific mechanisms. First, the senescent like phenotype induced by RRx-001 may, in turn, induce an increased erythrophagocytosis and the consequent ferroptosis in the more phagocytic M2 macrophages, thereby shifting the balance to the antitumor M1 phenotype as described by Youssef et al. [93] or by Dai et al. [94]. Secondly, the heme and Fe^++^ released via hemolysis of adhered RBCs may be taken up by tumors and induce ferroptosis.

Enhanced RRx-001-treated RBC adhesion to endothelial cells was in fact demonstrated, especially in the presence of TNF-α and hypoxia, this supporting the belief that the adhesion couple was PS-PSR.

In relation to oxidative stress and its involvement in the development of anticancer therapeutic strategies, it is worth mentioning the role of ion channels, transmembrane proteins regulating the permeability of cell membranes to ions. Oxidative stress occurs when there is an imbalance between the production of free radicals and the defense mechanisms implemented by antioxidant molecules. In physiological conditions, cells produce ROS that function as second messengers for intracellular signaling. The redox balance is maintained by various endogenous antioxidants. These include small molecules and enzymes such as glutathione, alpha-lipoic acid, catalase, superoxide dismutase, glutathione peroxidases. Natural antioxidant compounds can also be obtained from the diet, such as beta-carotene, ascorbic acid, and tocopherol. Antioxidant compounds counteract oxidation thus preventing cellular damage caused by free radicals. Experimental studies have demonstrated the ability of antioxidants to prevent skin cancer induced by UV exposure [95]. When ROS production exceeds the ability of antioxidant mechanisms to clear them thus determining an excessive increase of their concentration, cellular and tissue damage occurs. 

Ion channels have an important role in the regulation of cell migration, cell cycle progression, and proliferation. The genetic alterations occurring in cancer cells also involve the expression and/or activity of ion channels. Ionic signaling regulates innate and adaptive immune cells functions, extracellular matrix formation and tumor vascularization, thus influencing tumor microenvironment. ROS production induces post-translational modifications of ion channels, thus altering intracellular signaling pathways. Depending on the type of modification, oxidative stress can enhance or impair ion channels activity, thus promoting or attenuating the progression of tumors such as melanoma. As ion channels are primarily expressed in the plasma membrane, they represent promising targets for the development of anti-cancer therapeutic strategies. In a recent review, Remigante and co-authors thoroughly discussed the role of ion channels as therapeutic targets in melanoma [96]. 

### 3.3. The Role of the Red Blood Cell in Other Diseases

Central retinal vascular occlusion: Central retinal vascular occlusion (CRVO) is the second most common cause of vision loss [97,98] and has been associated with enhanced red blood cell adhesiveness to the retinal vasculature [13] where PS and PSR exposure have been implicated in the adhesiveness [13,98]. This is consistent with the findings of local inflammatory conditions causing oxidative stress that is known to promote PS and PSR exposure [9]. Hirabayashi et al. [98] report proinflammatory (M1) macrophage activity and oxidative stress whose therapeutic mitigation led to beneficial results. Without such mitigation, classically activated M1 macrophages may release TNF-α that, along with a typically hypoxic state, also promotes PS and PSR exposure on RBCs and the microendothelium, respectively [15].

Polycythemia vera: Polycythemia vera (PV) is a chronic myeloproliferative disease in which arterial and venous thrombosis is a main cause of morbidity and mortality [99]. In polycythemia vera the muted JAK2 kinase causes phosphorylation of BCAM (CD239) which is a receptor for endothelial laminin-α5 [25,26,27], and this may well be a main source of RBC adhesiveness. On the other hand, Tan et al. [100] report of observations of erythrocyte derived PS exposing “microparticles” (MPs); these MPs were described as vesicles in the size range 0.1–1 µm in diameter and as being “procoagulant”, and hence a likely contributor to the hypercoagulable state in PV. The pathway for such adhered RBCs to produce vesicles has been described by Asaro and Cabrales [2].

Diabetes: In diabetic retinopathy (DR), the breakdown of the blood–retinal barrier (BRB) results in vascular leakage and subsequent macular edema; this constitutes a major cause of vision impairment related to diabetes [101,102]. Importantly, diabetes causes oxidative stress, and elevated levels of oxidative stress contribute to the pathogenesis of diabetic vascular dysfunction, viz. that characteristic of DR [102]. The RBC may be the victim of ROS in the BRB, and then a contributor to unresolved ischemia and inflammation that leads to, inter alia, neovascularization, apoptosis of pericytes and astrocytes by the advanced glycation end-products (AGEs) that are produced [103]. Wautier et al. [104] had, in fact, hypothesized that prolonged RBC exposure to plasma hyperglycemia could cause AGE-like modification of RBC surface membrane proteins. This would enhance the adhesiveness of diabetic erythrocytes, via RAGE exposure, to the BRB endothelium. This would set the stage for RBCs contributing to further oxidative stress as per the paradigm described herein.

Alzheimer’s disease: Alzheimer’s disease (AD, besides affecting central nervous system, also impacts peripheral tissues and cells. Among them are also RBCs. 

## 4. Removal of Intracellular Inclusions: Siderocytes and Macrophages 

De Back et al. [40] describe the possible role of splenic macrophages in facilitating RBC vesiculation. They couch their hypothesis in the context of vesiculation being a mechanism for the removal of inclusions such as Heinz bodies (aggregates of denatured, oxidatively damaged hemoglobin) or malarial parasites [105,106]; this pitting function for malarial parasites has been further confirmed more recently, e.g., see Buffet et al. [107]. It is interesting that this basic type of mechanism was recognized over 66 years ago for siderocytes, i.e., RBCs with one or more granular inclusion containing ferric Fe [105]. The basic mechanism suggested in these cases was splenic vesiculation as hypothesized by Willekens et al. [108] for the loss of hemoglobin from circulating older RBCs. Macrophages have a pivotal role in these processes, yet by a currently specified molecular mechanism [40]. A possibility is, however, that aged, senescent, or diseased, RBCs that expose adhesion molecules may find macrophages a suitable adhesion substrate. This then brings into play our paradigm [1,2] that leads to vesiculation and then, with sufficient time, to later lysis and ghost formation; for RBCs that are merely aged, siderocytes, but not senescent or diseased, the process may terminate with vesiculation hence providing the self-protective mechanism described by Willekens et al. [108,109]. We recall the findings of Asaro and Cabrales [1,3] that sufficient time is required to proceed along their vesiculation–lysis–ghosting pathway, and so adhesion strength may be a vital factor. The videomicroscopy of MacDonald et al. [39] indeed shows quite clearly that RBCs routinely become “hung-up” in the splenic sinus, for times of only seconds that may induce vesiculation but insufficient to lead to lysis or ghost formation. Clearly, there is much to learn about the details of this adhesion–hemolysis–ghost formation paradigm that mediates RBCs life, death, and the RBCs role(s) in a wide range of diseased states.

## 5. Discussion and Future Prospects

The paradigm discussed herein, that is, of adhered red blood cells subject to modest shear flows leading to vesiculation, hemolysis, and ghost formation, provides a unifying scenario of the RBCs’ road to clearance directly coupled with the role(s) RBCs play in disease progression and—in particular cases—the role(s) they may play in disease regression. This suggests that the details of RBC adhesiveness may provide a diagnostic set of tools for assessing, and even designing, therapeutic strategies and methods.

There is, however, much to be learned concerning the role(s) played by molecular kinase and proteinase actors as well as by the triggering of mediating ion channels, e.g., Piezo1, in this time dependent process. In particular, sorting out their desperate time scales that mediate their action, as well as their relative importance is required to fully anticipate the expected role(s) that RBCs may play on disease progression; this understanding is clearly required for the design of therapeutic strategies and methods.

**Figure 4:**
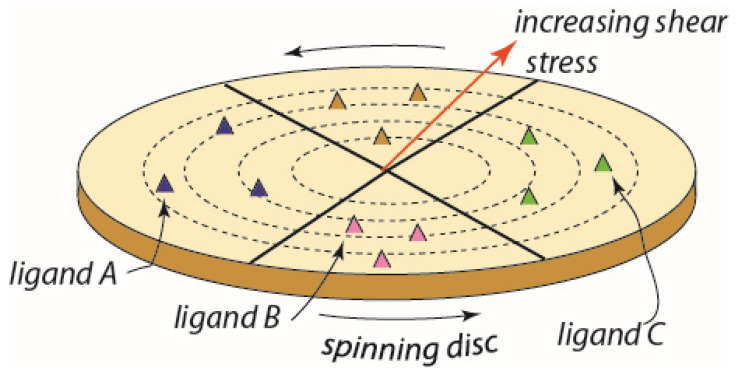
A conceptual scheme for a high throughput device based on a functionalized spinning disc. In this case, the sectors may contain different ligands and a gradient of shear stress is generated along the disc radius.

Figure 4 is meant to provide a conceptual approach to such studies-and perhaps to a diagnostic methodology-in that it illustrates a high-throughput design to assess both adhesive strength of RBCs to specific ligands and may be used to assess the potential for vesiculation, hemolysis, and ghosting. 

The essential idea is to subject red blood cells to a wide range of physiological level shear flow stresses upon substrates containing a range of specific adhesion ligands. In this manner, both adhesive strength and specificity for various ligands would be documented in a high-throughput manner. Moreover, such assays would be made for RBCs that were in various states including, inter alia, age, condition of oxidative stress and hypoxia as well as energy (i.e., ATP) depletion and conditions of blood storage, i.e., those leading to the senescent conditions as may exist in the conditions described above. Moreover, the RBCs of patients known to have any of a variety of hereditary anemic disorders, or diabetes, may be cataloged with respect to their adhesiveness to specific ligands and potential to follow our pathway to vesiculation–hemolysis–ghosting. 

The spinning-disc method is based on the classical fluid mechanics analysis of laminar flow near a rotating disc of Karman [110] in 1921 followed by Cochran [111] in 1934 and hence is quite well established and discussed in the mechanics literature. It has been used in a considerable range of studies on cell adhesion, e.g., see refs. [112,113,114]. In particular, Fuhrman and Engler [113] presented a concise description of the nature of the flow field acting on adhered cells and demonstrated that fluid flow and the shear stresses exerted on adhered cells are readily described, measured, and interpreted with respect to cell detachment and/or cell spreading. Thus, we propose it may provide a readily adapted methodology to explore specificity to a range of ligands that are known to bind specific receptors. 

Together with microfluidic devices, it would be useful to evaluate this pathway using complementary analyzes throughout the process, particularly single-cell omics and/or spatial omics. Advanced technologies that make it possible to perform mono-omics measurements at the single-cell level, such as single-cell DNA sequencing and RNA sequencing, based on the physical separation of RNA from DNA have been developed. More recently, methods to assess single cell epigenomics and transcriptomics characteristics in parallel have been developed. These methods give information on DNA methylation, histone modifications and interaction with transcription factors, in parallel with transcriptome. The multi-omics approach provides data that supply an in-depth analysis of cells, uncovering epigenome and transcriptome changes in physiological and pathological conditions. Next to single-cell omics, a spatial multi-omics approach can be used to evaluate different molecular analytes at the subcellular level within their native tissue context. Spatial multi-omics analysis is generally performed on fixed fresh-frozen or formalin-fixed paraffin-embedded tissue sections by combining spatial mono-omics methods, such as array-based spatial transcriptomics, in situ sequencing, and mass spectrometry imaging [115]. The combination of several technological approaches would provide more complete information useful for understanding RBCs adhesiveness in health and disease. That is, it may be insufficient to merely document one measure of RBC adhesiveness, or another, as is done in various protocols, but instead necessary to fully document the time scales of adhesiveness and the outcomes of their adhesiveness vis-a-vis hemolysis and ghost formation. On the one hand, it is the pathway of vesiculation–hemolysis–ghosting that releases the RBCs ROS, and yet it is the state of the RBC and its skeletal-membrane integrity that contributes vitally to the pathway.

## Data Availability

Not applicable.
